# A single dose of estrogen during hemorrhagic shock protects against Kidney Injury whereas estrogen restoration in ovariectomized mice is ineffective

**DOI:** 10.1038/s41598-020-73974-5

**Published:** 2020-10-14

**Authors:** Marie Buléon, Mélodie Cuny, Jimmy Grellier, Pierre-Yves Charles, Julie Belliere, Audrey Casemayou, Jean-François Arnal, Joost-Peter Schanstra, Ivan Tack

**Affiliations:** 1 Institute of Cardiovascular and Metabolic Disease, Institut National de la Santé et de la Recherche Médicale (INSERM), U1048, Toulouse, France; 2 Université Toulouse III Paul-Sabatier, Toulouse, France; 3 Service des Explorations Fonctionnelles Physiologiques, CHU de Toulouse, 1 avenue Jean Poulhès, 31 059 Toulouse Cedex 9, France

**Keywords:** Acute kidney injury, Animal disease models

## Abstract

The protective effect of estrogens against chronic glomerular diseases is admitted but remains debated during acute kidney injury (AKI). Using a model of resuscitated hemorrhagic shock in C57/Bl6 female mice, this study evaluated at 1 and 21 days the renal effect of (1) endogenous estrogen, using ovariectomized mice with or without chronic estrogen restoration, or (2) exogenous estrogen, using a single administration of a pharmacological dose during shock resuscitation. In both ovariectomized and intact mice, hemorrhagic shock induced epithelial cell damages (assessed by KIM-1 renal expression) with secondary renal fibrosis but without significant decrease in GFR at day 21. Ovariectomy with or without estrogen restoration have no significant effect on renal damages and dysfunction. This lack of effect was associated with a marked (> 80%) reduction of total kidney GPR30 expression. By contrast, a single high dose of estradiol in intact mice reduced renal KIM-1 expression by 2/3, attenuated the severity of cell death related to pyroptosis, and prevented the increase of fibrosis by 1/3. This provides a rationale to investigate the benefits of a single administration of estrogen or estrogen modulators during acute kidney injuries in males. Furthermore, the cost/benefit ratio of such administration should be investigated in Human.

## Introduction

Although estrogens are principally reproductive hormones, their receptors are widely expressed in the body and contribute to multiple non-reproductive effects that include cardiovascular actions, metabolic regulation, and tissue trophicity and repair^1^. Among non-reproductive organs, kidneys express one of the highest levels of estrogen receptors (ER), particularly ERα^2^. Paradoxically, descriptions of renal actions of endogen estrogen are sparse and appear mostly related to water, electrolyte homeostasis and glomerular matrix turn-over^3–6^. Data from the literature also supports the potential renal protective effect of endogen estrogen during Chronic Kidney Disease (CKD). Indeed, before menopause, CKD evolves more slowly in women than in men, particularly among patients with diabetes or IgA nephropathy^7–9^. The protective effect of endogen estrogen has been described in various experimental models of CKD: diabetic nephropathy, glomerulonephritis, and unilateral nephrectomy. This effect is generally lost in case of ovariectomy but in most cases, is restored with exogenous estrogen supplementation^10,11^.

Less is known regarding the potential renal protective effects during acute kidney injury (AKI). In humans, females appear to be protected against organ failure after major hemorrhage^12^. However, mechanisms of this gender dimorphism, that likely involve hormonal status, remain to be demonstrated in the absence of clinical trials^13^. Interestingly, pregnancy, a status with a higher estrogen impregnation, is associated with reduced trauma mortality when compared to matched non-pregnant women—a comparison that excludes a simple gender effect^14^. Conversely, a recent prospective study has shown that during septic shock, higher serum estradiol levels were associated with lower survival and a higher risk of AKI, but most patients in this study were males. Regarding experimental data, estrogen, either endogenous or exogenous, have been associated with significant benefits in various models of AKI such as cisplatin kidney toxicity^15^, renal ischemia reperfusion^16,17^ and cardiac arrest^18,19^. Hemorrhagic shock, a highly relevant situation in clinical practice, is often responsible for acute kidney injury that worsens the prognosis^20^. Among protective pharmacological interventions, administration of estrogen has been proposed in females and males alike. Despite the evidence of some benefits regarding cell protection and the modulation of inflammatory response^13^, the practical renal benefit (i.e. renal function and fibrosis) remains to be demonstrated. Furthermore, most works have not considered the distinction between estrogen restoration at physiological doses after ovariectomy and the administration of a high pharmacological dose of estrogen in intact animals.

We have previously developed a model of pressure-controlled resuscitated hemorrhagic shock in mice that exhibits a good level of anthropomorphism^21–23^. Based on this model, the present study was designed to evaluate the renal protective effect of estrogen in two situations: (1) restoration of estrogen impregnation in ovariectomized mice; (2) additional administration of a single bolus of estrogen at the time of resuscitation in intact female mice. We evaluated early renal damages and sequels. Regarding mechanisms, we focused on epithelial cell death related to pyroptosis that has been involved during ischemic AKI^24^ and that could contribute to inflammatory response and secondary fibrosis, potential targets for estrogen as shown in the brain^25^.

## Results

**Figure 1:**
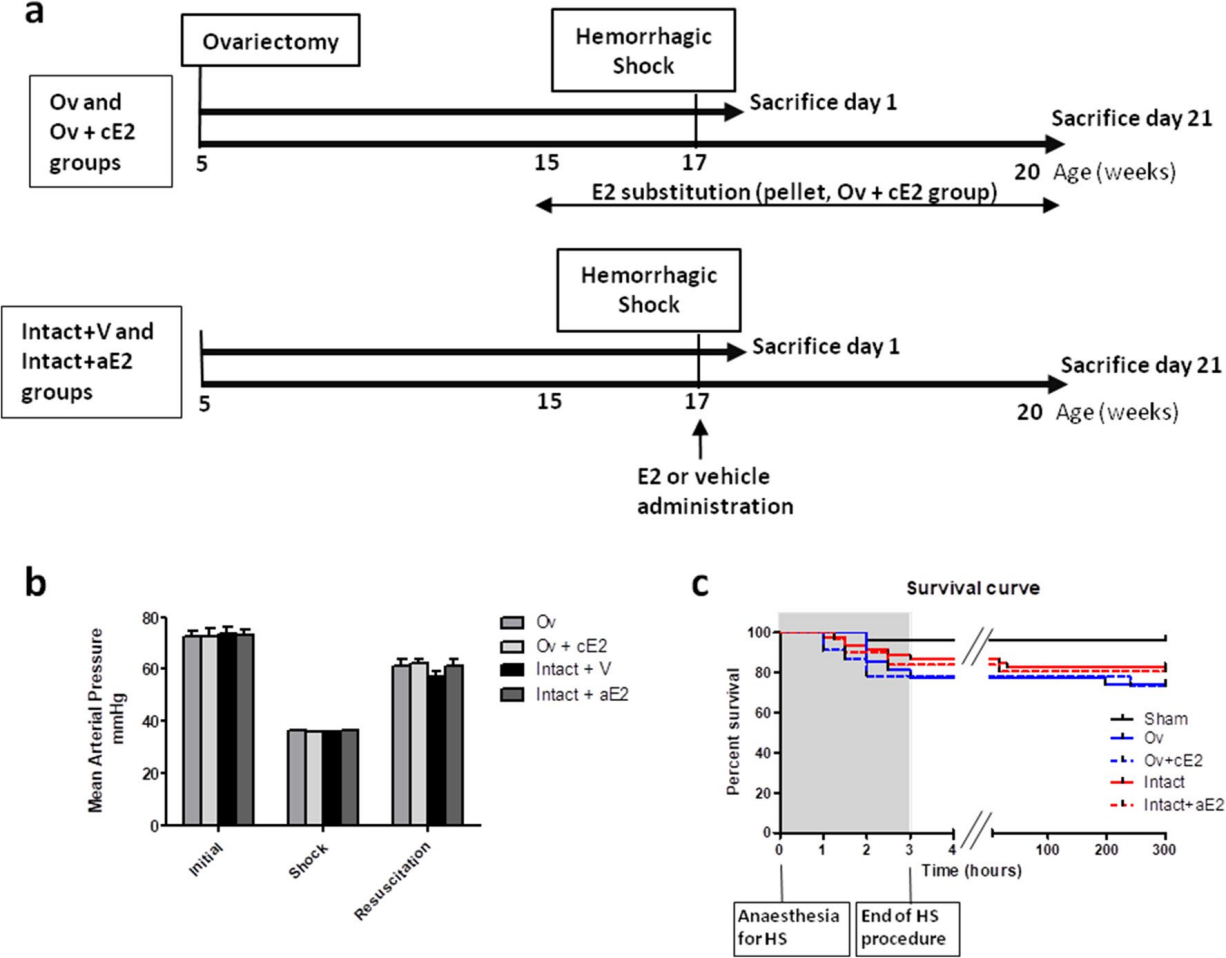
(**a**) Experimental design; (**b**) Mean arterial blood pressure (MAP) during hemorrhagic shock procedure (Initial = basal MAP before blood withdrawal, Shock = mean MAP during the 2 h of shock, and Resuscitation = mean MAP during infusion of blood and Ringer Lactate); (**c**) survival curve during and after hemorrhagic shock. *Sham: non-shocked mice; Ov: ovariectomized mice (n* = *19); Ov* + *cE2: ovariectomized mice with chronic estradiol substitution (n* = *16); Intact* + *V: Non ovariectomized mice receiving vehicle (n* = *24); Intact* + *aE2: intact mice receiving 25 µg of estradiol at the end of resuscitation (n* = *20).* For MAP, no significant difference between groups for each period, *P* < 0.001 between initial and shock and between shock and resuscitation for all groups. For mortality, there was no significant difference between shocked groups.

Protocol design is described in Fig. 1a.

### Effect of hemorrhagic shock procedure

Initial mean arterial blood pressure was not different between experimental groups (73.0 ± 1.1 mmHg, Fig. 1b). Ovariectomy (Ov group) and chronic E2 substitution (Ov + cE2 group) had no impact on basal blood pressure. During the hemorrhagic shock procedure (2 h), mean arterial blood pressure was 36.1 ± 0.1 mmHg, for a target of 35 mmHg, and was not different between groups. Mean blood volume removal necessary to reach this pressure was 0.7 mL and was not different between groups. Mean blood pressure during resuscitation (blood restitution and Ringer’s lactate infusion, approximately 20 min) was 59.6 ± 1.2 mmHg and was similar between groups. We therefore considered that the severity of the hemorrhagic procedure was similar between groups.

Death rates consecutive to hemorrhagic shock procedure were 17% in the intact shocked mice (Intact + V group), 26% in the Ov group, 25% in the Ov + cE2 group and 20% in the intact mice treated with a single administration of estradiol (Intact + aE2 group). These slight differences in mortality were not statistically different. As shown is Fig. 1c, most of the mortality resulted from mice that died during or immediately after the HS procedure.

### Renal impact of hemorrhagic shock

**Figure 2:**
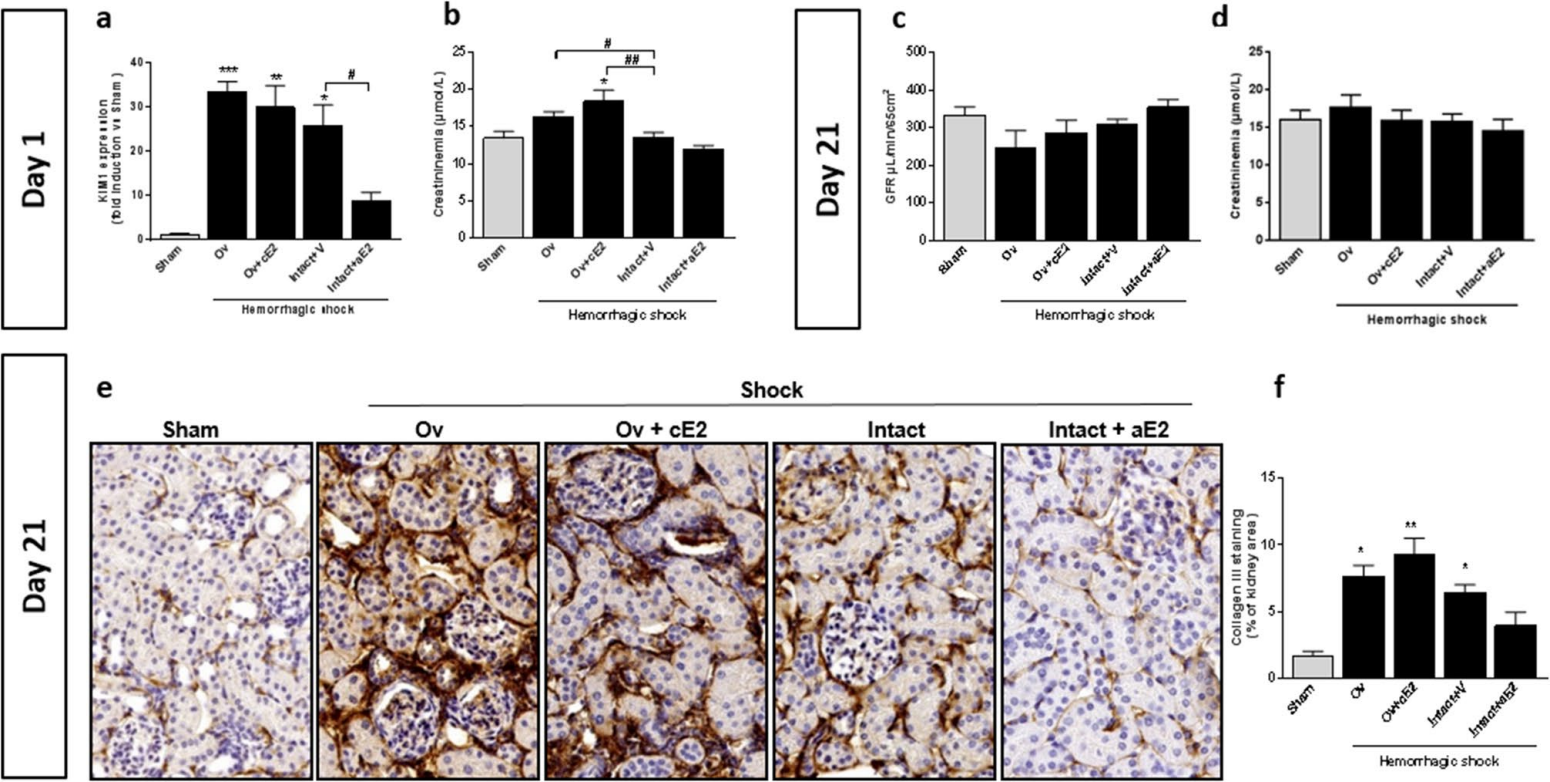
Renal impact at day 1 after hemorrhagic shock (**a**,**b**). (**a**) Tubular injury (KIM-1 mRNA expression, n = 6 for all groups), (**b**) Blood creatinine (n = 8 for sham and Intact, n = 7 for Ov, n = 6 for Ov + cE2 and n = 7 for Intact + aE2); Renal impact 21 days later (**c**–**f**). (**c**) Glomerular Filtration Rate measured by inulin clearance (n = 9 for sham and Intact, n = 6 for Ov, n = 5 for Ov + cE2 and n = 6 for Intact + aE2), (**d**) Blood creatinine (n = 6 for all groups), (**e**) Representative pictures of collagen III staining on kidney sections (magnification × 400, immunohistochemistry, counterstaining with hematoxylin), (**f**) Quantification of Collagen III expression (immunohistochemistry, n = 5 for each group). *Sham: non-shocked mice; Ov: ovariectomized mice; Ov* + *cE2: ovariectomized mice with chronic estradiol substitution; Intact* + *V: Non ovariectomized mice receiving vehicle; Intact* + *aE2: intact mice receiving 25 µg of estradiol at the end of resuscitation.* For KIM1 mRNA expression, relative mRNA expression levels were calculated using the ΔΔCtCt method, normalized to HPRT and β actin, and expressed as fold changes relative to the sham animals. Data are presented as mean ± s.e.m. *P < 0.05; **P < 0.01 and ***P < 0.001 versus sham; ^#^*P* < 0.05 and ^##^*P* < 0.01 for indicated comparison.

KIM-1 (Kidney-Injury-Molecule-1) was used as an early marker of acute tubular injury (Fig. 2a). One day after Acute Kidney Injury (AKI), KIM-1 was increased in all shocked mice. There was no significant difference between intact and ovariectomized mice for KIM-1 expression. By contrast, a single intravenous bolus of estradiol (25 µg) administered at the end of resuscitation in non-ovariectomized female mice significantly reduced the increase in KIM-1 after HS when compared to vehicle treated mice.

Creatininemia did not increase in intact shocked mice whereas a subtle increase was detected in ovariectomized mice, whether substituted with estradiol or not (Fig. 2b). Creatininemia remained unaffected by acute estradiol administration.

Twenty-one days after AKI, shocked mice recovered a normal GFR (Fig. 2c) that was not significantly different between groups, although it tended to be slightly lower in Ov group. Similarly, creatininemia was not different between groups. However, shocked animals had developed significant renal fibrosis, as assessed by immunohistochemical analysis of collagen III deposition (Fig. 2d,e,f). Fibrosis was slightly more pronounced in ovariectomized mice, whether they were supplemented or not and was lower than in Intact + V and Intact + aE2 mice.

Finally, the lack of endogenous estrogen did not worsen acute kidney injury and estrogen substitution in ovariectomized mice did not prevent the renal impact of hemorrhagic shock. By contrast a single administration of estradiol at the end of HS in intact mice protected against KIM-1 induction at day 1 and fibrosis development 21 days later.

### Uterus weight and estrogen receptor expression

**Figure 3:**
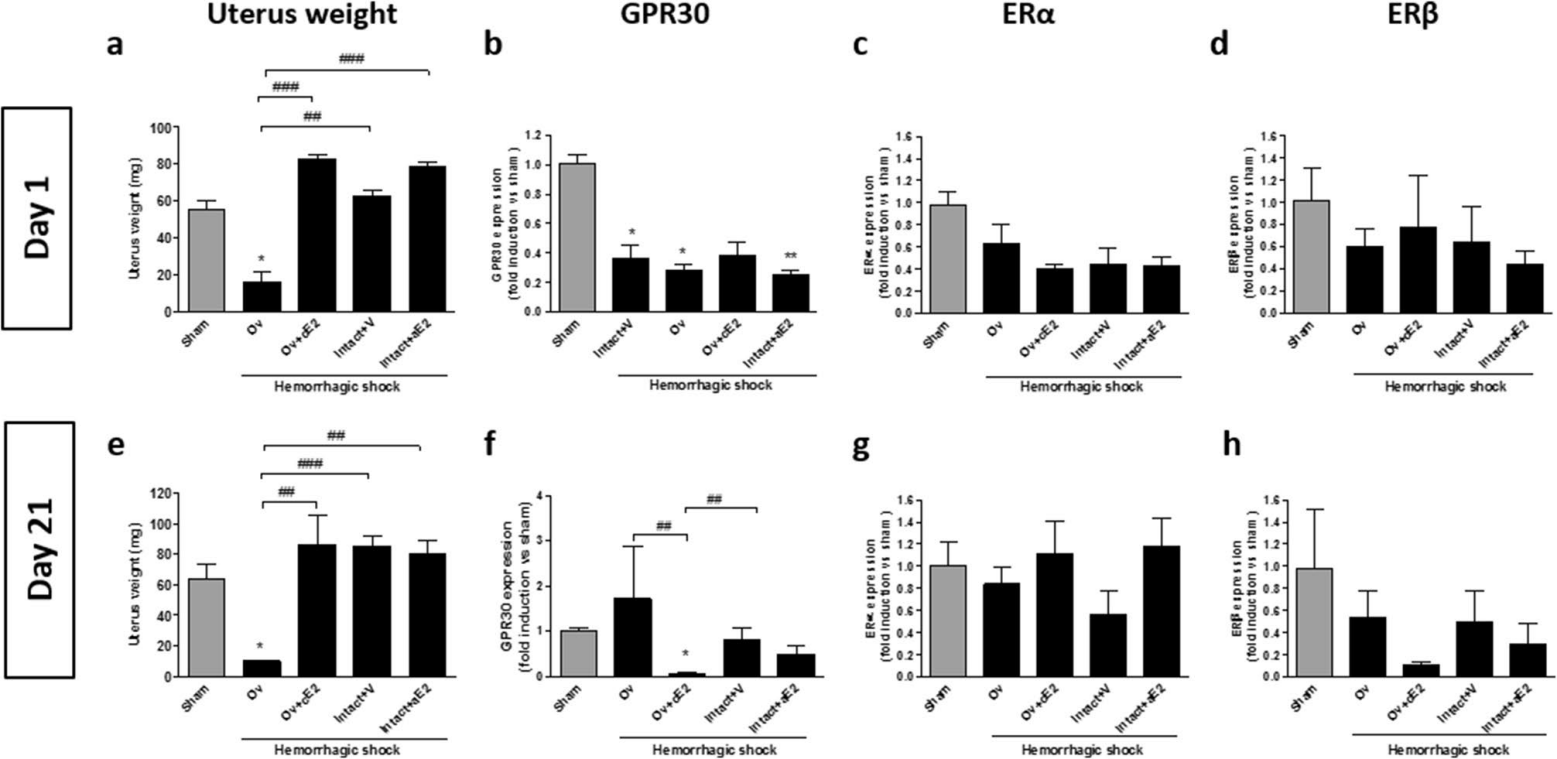
Estrogen receptors expression from whole kidney and uterus weight at day 1 (**a** to **d**) and day 21 (**e** to **h**) after hemorrhagic shock. (**a** and **e**) estrogen impregnation assessed by measure of uterus weight; (**b**,**f**) expression of GPR30; (**c**,**g**) expression of estrogen-receptor α; (**d**,**h**) expression of estrogen-receptor β. *Sham: non-shocked mice; Ov: ovariectomized mice; Ov* + *cE2: ovariectomized mice with chronic estradiol substitution; Intact* + *V: Non ovariectomized mice receiving vehicle; Intact* + *aE2: intact mice receiving 25 µg of estradiol at the end of resuscitation. N* = *6 in each group.* Relative mRNA expression levels were calculated using the ΔΔCtCt method, normalized to HPRT and β actin, and expressed as fold changes relative to the sham animals. Data are presented as mean ± s.e.m. *P < 0.05 versus sham group; ^##^*P* < 0.001 and ^###^*P* < 0.001 for indicated comparison.

In order to verify the absence of estrogen impregnation in ovariectomized mice, we measured the weight of the uterus at days 1 and 21. Uterus weight showed a strong decrease in the Ov group compared to all other groups. In contrast, estrogen restoration using pellets resulted in a uterus weight similar to that of intact mice (Fig. 3a,e).

The expressions of estrogen receptors ERα, ERβ and GPR30 in whole kidney extracts were studied by RT-PCR. GPR30 mRNA expression was significantly reduced in all shocked groups one day after HS and remained significantly lower than the sham group in the Ov + cE2 group at day 21, whereas other groups were similar to the sham (Fig. 3b,f). ERα mRNA expression seemed to have decreased after HS (at day 1) in all shocked animals compared to the sham, but this difference was not significant (Fig. 3c). At day 21, ERα expression was similar in all groups (Fig. 3g). ERβ expression was quite heterogeneous, but values were similar in all groups for both day 1 and day 21 (Fig. 3d,h).

### Mechanisms involved in the renal effect of exogenous estrogen

We have explored mechanisms potentially involved in the mediation and modulation of damages, during the first 24 h following AKI and at the stage of tubular repair (3 weeks). Regarding cell death, programmed cell death apoptosis and pro-inflammatory cell death pyroptosis were studied since both have been described during renal ischemic injuries.

#### Apoptosis

**Figure 4:**
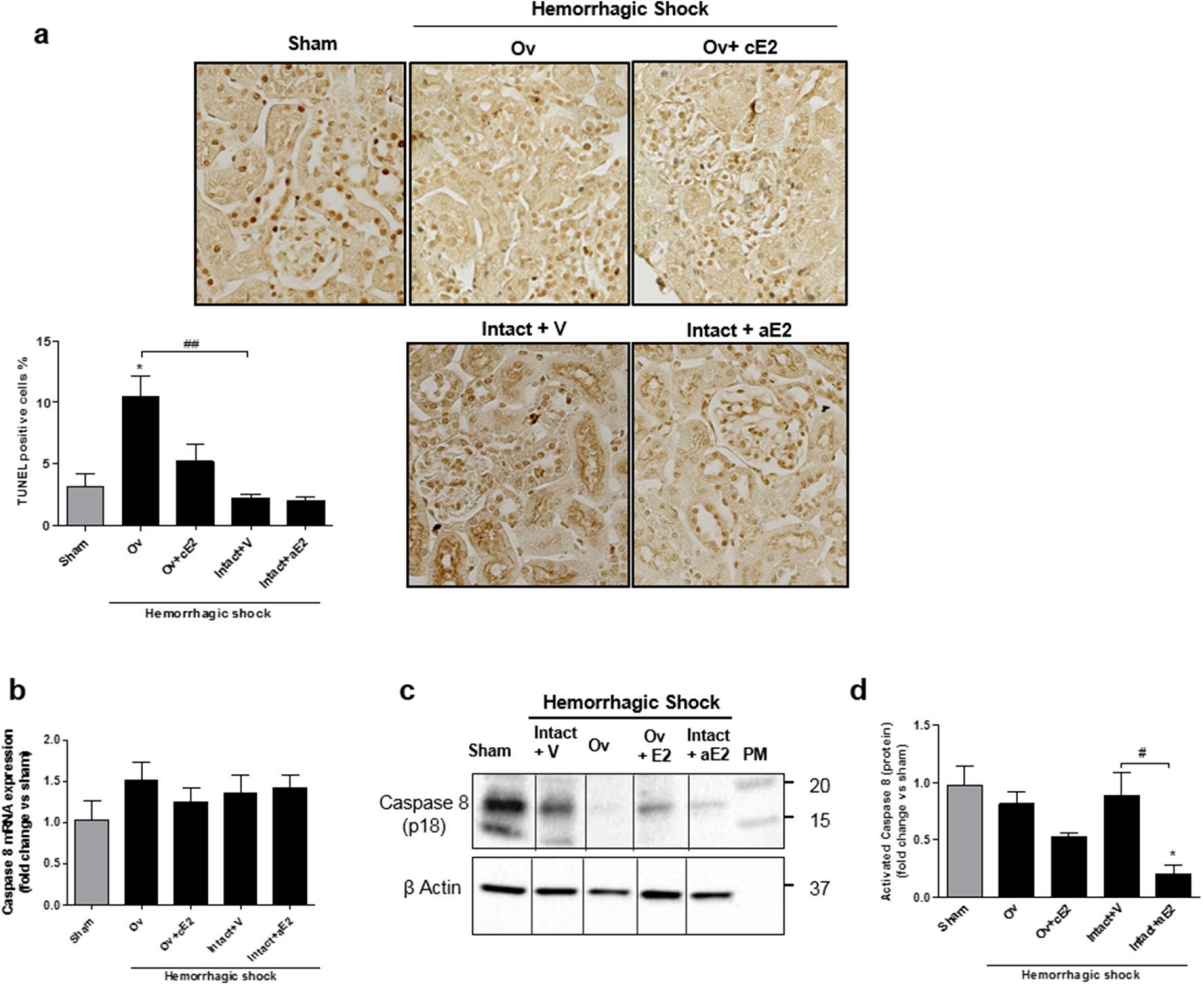
Cell death at day 1. (**a**) picture of renal cortex stained by TUNEL method (magnification X800*)* and quantification of stained nuclei*, n* = *6 for all groups.* Apoptosis at day 1 (**b** to **d**): (**b**) Caspase 8 mRNA expression (from whole kidney); (**c**) Western Blot of the cleaved-form of caspase 8; (**d**) quantification of the expression of cleaved-form of caspase 8 from 4 independent Western-Blot. *Sham: non-shocked mice; Ov: ovariectomized mice; Ov* + *cE2: ovariectomized mice with chronic estradiol substitution; Intact* + *V: non ovariectomized mice receiving vehicle; Intact* + *aE2: intact mice receiving 25 µg of estradiol at the end of resuscitation.* Relative mRNA expression levels were calculated using the ΔΔCtCt method, normalized to HPRT and β actin, and expressed as fold changes relative to the sham animals. Data are presented as mean ± s.e.m. *P < 0.05 versus sham; ^#^*P* < 0.05 and ^##^*P* < 0.01 for indicated comparison.

Programmed cell death was quantified by TUNEL, which stains degraded nuclei (Fig. 4a). Paradoxically, the sham group exhibited cell death, suggesting that the anesthesia and surgery performed on these animals had detrimental effects to some extents. Cell death following shock was increased in ovariectomized mice compared to intact. This increase appeared attenuated in Ov + cE2 mice. Cell death in both the control and estradiol groups was similar to that of the sham.

The contribution of apoptosis was checked using caspase 8 protein expression, the initiator and specific marker of the classical apoptosis pathway. In the sham and shocked groups, the expression of caspase 8 mRNA was not different (Fig. 4b). However, a differential expression of its active cleaved form (p18) studied using WB (Fig. 4c,d), was observed. The sham group already presented a high expression of this active form that was not different from that of the Intact + Vehicle and ovariectomized shocked mice. By contrast, the expression of activated caspase 8 was significantly lower in estradiol-treated mice.

#### Pyroptosis

**Figure 5:**
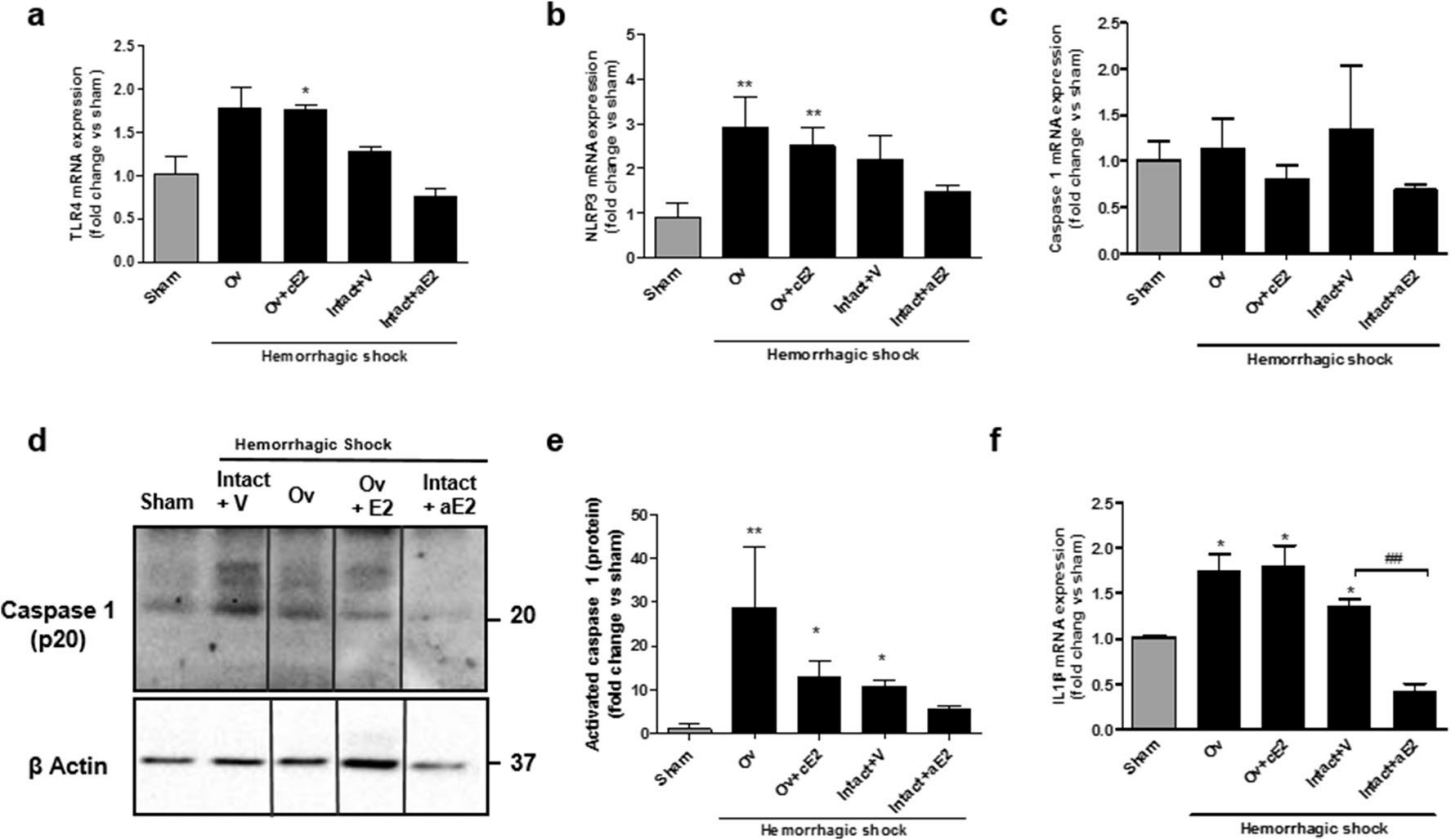
Inflammasome and pyroptosis one day after hemorrhagic shock. (**a**) TLR4 mRNA expression; (**b**) NLRP3 mRNA expression; (**c**) Caspase 1 mRNA expression; (**d**) Western Blot of the cleaved-form of caspase 1; (**e**) quantification of the expression of cleaved-form of caspase 1 from 4 independent Western-Blots. (**f**) IL1β mRNA expression. *N* = *6 per groups for mRNA and n* = *4 per group for protein. Sham: non-shocked mice; Ov: ovariectomized mice; Ov* + *cE2: ovariectomized mice with chronic estradiol substitution; Intact* + *V: non ovariectomized mice receiving vehicle; Intact* + *aE2: intact mice receiving 25 µg of estradiol at the end of resuscitation.* Relative mRNA expression levels were calculated using the ΔΔCtCt method, normalized to HPRT and β actin, and expressed as fold changes relative to the sham animals. Data are presented as mean ± s.e.m. Data are presented as mean ± s.e.m. *P < 0.01, **P < 0.001 versus sham; ^##^*P* < 0.01 for indicated comparison.

We further examined markers for pyroptosis. The TLR4-induced inflammasome cascade (TLR4 – NLRP3 – caspase 1 – IL1β) appeared to be recruited during shock in intact and ovariectomized animals even if the changes in TLR4 and NLRP3 mRNA expression did not reach significance because of individual disparity (Fig. 5). In the group that has received the bolus of estradiol (Intact + aE2), TLR4 and IL1β expression was significantly reduced with a similar trend for NLRP3. The caspase 1-cleaved form was increased by HS in intact mice in the vehicle group. The bolus of estradiol partially prevented this increase and results were not different from that of the sham group.

**Figure 6:**
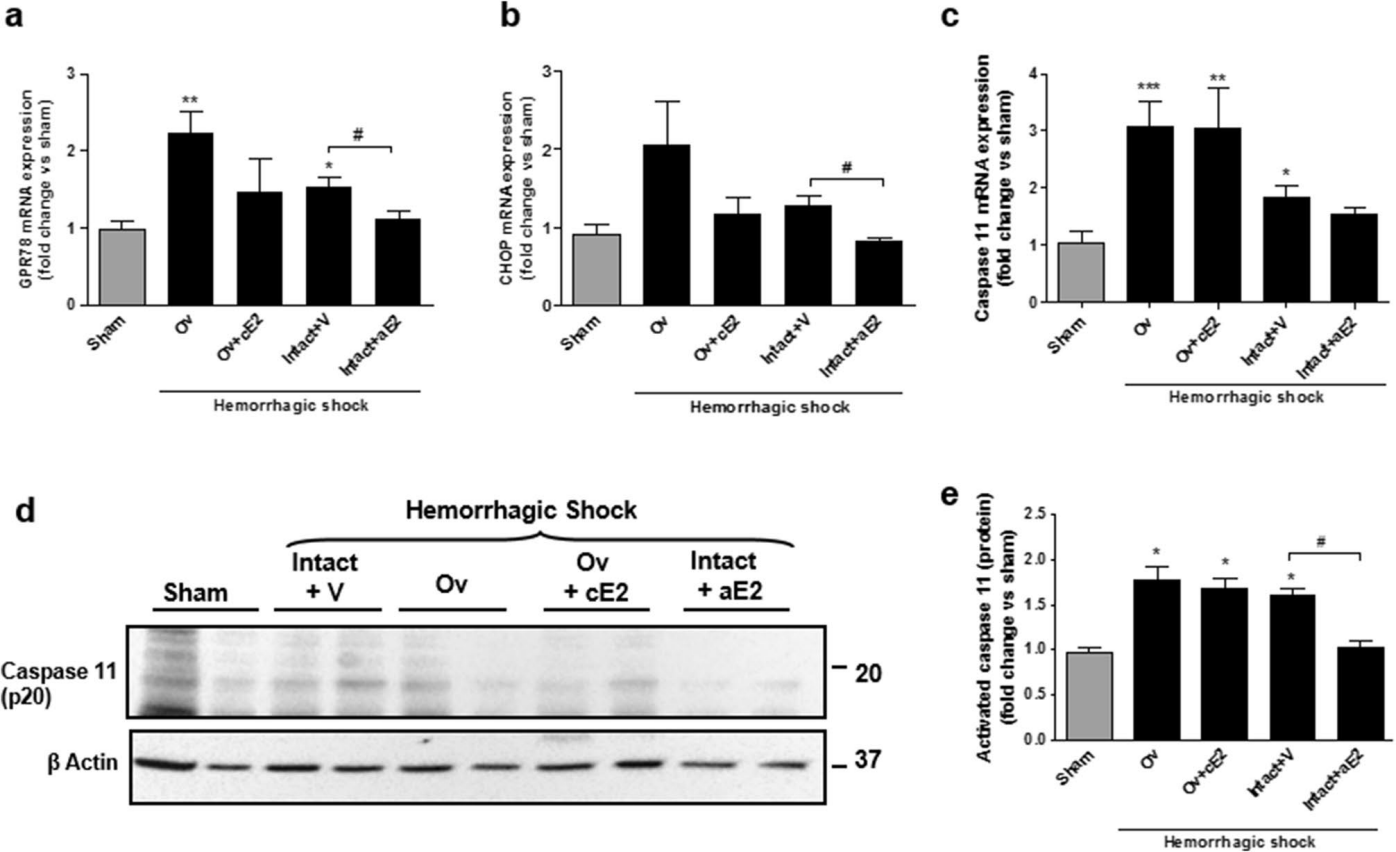
Endoplasmic reticulum stress and pyroptosis at day 1. (**a**) GPR-78 mRNA expression; (**b**) CHOP mRNA expression; (**c**) Caspase 11 mRNA expression; (**d**) Western Blot of the cleaved-form of caspase 11; (**e**) quantification of the expression of cleaved-form of caspase 11 from 4 independent Western-Blots. *N* = *6 per groups for mRNA and n* = *4 per group for protein. Sham: non-shocked mice; Ov: ovariectomized mice; Ov* + *cE2: ovariectomized mice with chronic estradiol substitution; Intact* + *V: non ovariectomized mice receiving vehicle; Intact* + *aE2: intact mice receiving 25 µg of estradiol at the end of resuscitation.* Relative mRNA expression levels were calculated using the ΔΔCtCt method, normalized to HPRT and β actin, and expressed as fold changes relative to the sham animals. Data are presented as mean ± s.e.m. *P < 0.01, **P < 0.001 and ***P < 0.001 versus sham; #*P* < 0.05 for indicated comparison.

Pyroptosis activation involves endoreticular stress. Expression of GPR78 and the transcription factor CHOP produced by the endoplasmic reticulum in case of stress were increased in shocked mice, particularly in the Ov group (Fig. 6). Conversely, these expressions remained similar to that of sham mice in the acute estradiol group. Caspase 11 is specific to the pyroptosis pathway and its synthesis is activated by CHOP. Caspase 11 mRNA expression was studied using qPCR and its active form (p20) was assessed by WB (Fig. 6c–e). Caspase 11 mRNA expression was increased in all shocked groups but the intact + aE2 group in which the expression was similar to the sham group. Activated caspase 11 expression was similarly modified by shock and by acute administration of E2. Particularly, activated caspase 11 in the intact + aE2 group was significantly decreased when compared to intact + vehicle and appeared similar to the sham group.

## Discussion

The key findings from this study are that during hemorrhagic shock in female mice, i) endogenous estrogens do not reduce death rate, acute kidney injury intensity, and secondary renal fibrosis, whereas ii) a single high-dose administration of estradiol during resuscitation in non-ovariectomized shocked mice exerts a protective effect associated with a reduced recruitment of renal pyroptosis pathway.

Endogenous estrogens are considered nephroprotective during chronic kidney disease^7–9^. This assumption is driven by both epidemiological data obtained before and after menopause in women and experimental data obtained in female rodents when comparing intact versus ovariectomized mice with or without estrogen restoration. These promising results are challenged by conflicting results in humans^26^. In particular, long-term estrogen treatment after menopause may negatively affect glomerular filtration rate in women with previous renal damages^27^.

During acute kidney injury, the benefits of estrogen also appear encouraging even if sparse and disparate. Several studies suggest that estrogen could limit renal damages in a number of models of AKI, including cardiac arrest, ischemia–reperfusion and cisplatin toxicity^15,18,28^. In these investigations, estradiol was administrated at a high dosage, most often in male (mice or rat) or ovariectomized female rodents. These studies had focused on early consequences regarding renal histology or function but not on delayed renal repair or sequels.

AKI induced by hemorrhagic shock in mice is a potentially anthropomorphic situation. To our knowledge, renal effects of estrogen during hemorrhagic shock in female rodents have not been studied yet, probably because these models are demanding. We have previously described a model of resuscitated hemorrhagic shock in female mice^23^ that was associated with approximately 20% death, acute kidney damage early in the protocol and renal functional recovery after 21 days with moderate renal fibrotic sequels. Herein, we used this model to establish the renal effects of: i) ovariectomy and of estrogen restoration; ii) an acute dose of estradiol given at the time of resuscitation in mice with intact gonadic functions. In the present study, the effect of a bolus of estradiol in Ov mice has not been investigated in detail in order to limit group multiplicity and because preliminary studies had not shown any differences with the Ov + cE2 group.

In the present work, the loss of estrogen impregnation resulting from ovariectomy in adult mice only slightly worsened the functional impact of hemorrhagic shock and had no significant effects on kidney histology. Additionally, the group of ovariectomized mice that received a pellet-based estradiol supplementation at 15 weeks and that was submitted to the shock 2 weeks later was not protected from renal impact despite the obvious effect of estradiol assessed by uterus weight determination^29^. The dose of estrogen we used was slightly supraphysiologic and completely prevented uterus atrophy. Such a dose has been shown to exert a protective effect in a model of skin flap necrosis in mice^30^. By contrast, our results suggest that endogenous estrogens in adult female mice do not play a noticeable protective role against acute hemodynamic injury. Of interest, estrogen receptors expressions (GPR30, ERα and ERβ) were not significantly altered in ovariectomized mice when compared to sham mice or intact shocked mice. The only significant difference (at day 21) was a marked and persistent decrease in GPR30 mRNA expression in Ov + cE2 mice. The down-regulation of GPR30 could have contributed to limiting the protective effect of estrogen in the Ov + cE2 group since transmembranous GPR30 receptor appeared to contribute to estradiol-related vascular protection^31^. The lack of protective effects of estrogen restoration in our study contrasts with the beneficial renal effect observed in a previous work that used a model of ischemia reperfusion in mice^17,28^. However, the dose of estrogen used in the above study was almost twenty times higher than in the present work. Thus, a differential action of such doses on ERα and ERβ receptors could have contributed to the beneficial effects of estradiol on this model. Such dose effect has already been described in the kidney^6^. Finally, the benefit observed in ovariectomized mice with high doses were quite similar to what we have observed with a single bolus of a high dose of estradiol in non-ovariectomized mice. Another explanation for the lack of efficiency that we have observed with a lower and more physiological dose could be that either the ovarian nephroprotective effect results from a more complex hormonal equilibrium, or that the delay in estradiol restoration following ovariectomy was too long, thus leading to an irreversible loss of function of this hormonal system despite persistent ERα and ERβ expression^32–34^. Complementary work is required to determine if the time interval between the loss of ovarian function and estradiol supplementation could explain the ineffectiveness of estrogen restoration.

The administration of a single bolus of estrogen at a high dose has been studied in male rats for which it was effective in improving the survival from hemorrhagic shock^35^. To our knowledge, the potential benefit of such a single dose has not been studied in non-ovariectomized female mice. Nonetheless, estradiol concentration can reach high values during a female’s life, particularly during pregnancy^29^. In this case, high concentrations of estrogen result in additional effects that could, for instance, contribute to the high tolerance of pregnant female during postpartum hemorrhage^14^. This prompted us to explore the consequences of an acute administration of estrogen in non-ovariectomized mice at the time of resuscitation in our model of AKI. The amount of estradiol used for the bolus in the present study was largely supra physiological. However, it was within the range of single doses used in previous studies^18,19,36^. The bolus of estradiol was able to limit the initial renal impact of hemorrhagic shock as assessed by KIM-1 expression. Three weeks later, the increase in fibrosis assessed by Sirius red staining was also reduced when compared to non-estradiol-treated shocked animals. However, the mild renal impact of hemorrhagic shock in our model using C57Bl6 strain made it difficult to determine if the protective effect of estradiol that we observed was clinically relevant. Nevertheless, this effect allowed us to examine the potential mechanisms of estrogen renal protection in this model of AKI.

The single bolus of estradiol partly prevented the rise in KIM-1 mRNA expression following shock. Total cell death assessed by TUNEL was slightly lower. Moreover, the mechanism of cell death appeared to have been shifted from a pyroptosis pathway profile to the more classical apoptosis pathway. At the same time, inflammasome response was also modified.

It has been recently shown that the pyroptosis pathway could be activated in epithelial renal cells^24^. This pathway is particularly immunogenic, resulting in a pronounced inflammatory response^37,38^. First identified in macrophages where it results from an activation of TLR4, pyroptosis appears possible in all cells through the activation of DAMPS (Damage / Danger-Associated Molecular Patterns)^39,40^ or directly through endoplasmic reticulum stress. Endoplasmic reticulum stress is a key early event of cellular aggression, which may lead to cell death by pyroptosis via the activation of the transcription factor C/EBP Homologous Protein (CHOP). This activation of CHOP induces, in turn, that of caspase 11 and the recruitment of inflammasome. In our study, hemorrhagic shock in female mice appeared to recruit pyroptosis pathways whereas in mice that received the bolus of estradiol, activation of that pathway was largely prevented. It is possible that the bolus of estrogen given during resuscitation modulated endoplasmic reticulum stress, possibly via GPR30, thus impacting cell death pathways and the resulting inflammation.

Neither suppression of endogenous estrogen production by ovariectomy nor restoration of estrogen impregnation using the prolonged administration of a physiological dose of estradiol had any significant impact in our model of hemorrhagic shock. In contrast, the present work provides evidences for a renal protective effect of a pharmacological dose of estradiol when acutely given at the time of resuscitation in the same model with intact ovarian functions. Whereas post-ovariectomy estrogen restoration largely questions about timing of restoration, dose and duration, the benefit of an immediate and large amount of estrogen during hemorrhagic shock suggests the recruitment of other supra-physiologic effects of this hormone^41^. Whether additional estradiol administration would further improve even more kidney protection has not been studied here. This limitation will require further work. However, the interest in the single administration of estrogen at the time of aggression relies in a lower risk of adverse events, potentially allowing their extended use during hemorrhagic shock. Even though morphological and functional damages were moderate in our model, this provides a rationale to extend the investigation regarding the potential protective effects of a single administration of estrogen on survival and renal impact in human.

Finally, whereas estrogen deprivation or restoration has minor effects, a single bolus of estradiol ameliorates renal injury in hemorrhagic shock in female mice. Further experiments should test the hypothesis in males. The results also support the study on the role of estradiol in human patients with hemorrhagic shock.

## Materials and methods

### Experimental model

Adult C57BL/6J female mice were purchased from Janvier Labs (Le Genest Saint Isle, France) and housed in a pathogen-free, temperature-controlled environment with a 12-h/12-h light/dark photocycle, as described^42^. Animals had free access to food and tap water. Animal experimentations were performed according to the national and institutional animal care and ethical guidelines and were approved by the animal care and use committee UMS US006/INSERM (Toulouse, France).

Protocol design is described in Fig. 1a. We studied the consequence of hemorrhagic shock (HS) in both: (1) endogenous estrogen depletion (by ovariectomy) with or without chronic restoration by subcutaneous estradiol pellets; (2) A single acute administration of estrogen in intact mice. For this purpose, 5 groups of mice were used: (1) Ovariectomized mice with HS (Ov); (2) Ovariectomized mice with subcutaneous estradiol pellets (0.1 mg E2, Innovative Research of America, releasing 80 µg/kg/day), with HS (Ov + cE2); (3) Intact mice with HS (Intact + V); (4) Intact mice treated with a single administration of 1 mg/kg of estradiol at the end of HS procedure (Intact + aE2); (5) Sham-operated mice.

One part of the mice was euthanized 1 day after HS procedure and the remaining part 21 days after HS.

### Ovariectomy

At the age of 5 weeks, mice from the Ov and Ov + cE2 groups were anesthetized with intraperitoneal injection of ketamine 125 mg/kg and xylazine 10 mg/kg. A small incision was made on the flank of the animal; the ipsilateral ovary was released and removed after ligation of the uterus. The muscle layers and skin were sutured separately. The same procedure was repeated on the other side. Non-ovariectomized females (Intact) underwent the same anesthetic and surgical protocol but without ovariectomy.

### Estradiol treatments

#### Chronic substitution

Two weeks before hemorrhagic shock, subcutaneous estradiol pellets (0.1 mg E2, Innovative Research of America, releasing 80 µg/kg/day) were placed at the nape of the neck.

#### Single administration

Cyclodextrin-encapsulated 17β-estradiol (β-estradiol-water soluble, Sigma E4389, 25 µg diluted in 0.2 mL of saline) or vehicle (0.2 mL of saline) was given intravenously at the time of the resuscitation from HS.

#### Hemorrhagic shock procedure

HS was performed as previously described^23^. Animals were anesthetized with ketamine and xylazine (125 mg/kg and 10 mg/kg, respectively) and intubated using an intra-tracheal cannula. Mechanical ventilation (9 mL/kg, 150 min^−1^) was carried out with a specific ventilator Minivent 845 (Hugo Sachs Elektronik, March-Hugstetten, Germany). Animal body temperature was continuously monitored and maintained to 37 °C. The left jugular vein was catheterized and anesthesia was maintained with ketamine (20 mg/kg/hour) until the end of shock. The femoral artery was catheterized and the catheter was connected to a pressure sensor and a blood pressure analyzer (IOX, EMKA technology) in order to monitor mean arterial blood pressure during all the procedure. Blood was withdrawn through the femoral arterial line until the mean arterial blood pressure reached 35 mmHg. Blood was stored in 0.15 mL of saline with heparin. Mean arterial blood pressure was maintained to 35 ± 5 mmHg for 2 h through successive blood withdrawals or replacements. At the end of that period, the blood previously stored and a Ringer's lactate solution (equal to the initial blood volume) were infused to provide appropriate fluid resuscitation. Sham-operated animals underwent the same anesthetic and surgical procedures, but neither hemorrhage nor fluid resuscitation was performed.

#### Renal function

At day 1, serum creatinine was measured on a Pentra 400 analyzer (HoribaMedical, Grabels, France) to provide an index of renal function. At day 21, GFR was measured using urinary clearance of inulin^43^. After anesthesia (Sodium Thiobutabarbital, Inactin, Sigma T133, 150 mg/kg) and tracheotomy, the jugular vein was catheterized for infusion of inulin (Cerb Laboratoire, France, 0.06 mg/min) and Gelofusine 4%. Another catheter was placed in the femoral artery for the monitoring of arterial blood pressure and collection of blood samples. Finally, an intravesical catheter was inserted for urine collection. At the end of surgery, 4 mg of inulin were injected intravenously. After 30 min of equilibration, urine was collected during one hour with a blood sample at mid-period. Plasma and urine inulin concentration were then measured. GFR was assessed by calculation of inulin clearance.

#### Gene expression quantification

Total RNA was isolated from mouse whole kidneys using Qiagen RN EasyPlus Mini kit (Qiagen, Valencia, CA). The sample RNA concentration was measured on a NanoDrop instrument (ND-1000 Spectrophometer) and RNA purity was determined by the A260/A280 and A260/A230 ratios as described^42^. For RT-PCR, 500 ng of total RNA were used for cDNA synthesis by Superscript II Reverse Transcriptase (Invitrogen, Life Technologies SAS). Biomark qPCR analysis was performed according to the manufacturer’s protocol (ADP37, Fluidigm, South San Francisco, USA). Water was loaded with primers as negative control. Primers had been previously tested to assure efficacy and rule out genomic DNA cross-reactivity. Relative mRNA expression levels were calculated using the ΔΔCtCt method, normalized to HPRT and β actin, and expressed as fold increases relative to the sham animals.

#### Protein expression

Tissue proteins exctracts (10 μg) were separated by electrophoresis in polyacrylamide gels 10% SDS-PAGE gels (Invitrogen, Life Technologies SAS) and electrophoretically transferred to nitrocellulose membranes (Hybond-ECL; Amersham). After 1-h incubation at room temperature in Tris-buffered saline, 5% milk, the blots were exposed to primary antibodies (Enzolife : caspase 1 mAb48^44^, caspase 8 mAb1G12^45^, caspase 11mAb4E11^46^ ; 1:500 in a solution Tris-buffered saline, 5% milk for 1 night at 4 °C). The primary antibodies were revealed using the corresponding rabbit or mouse peroxidase- conjugated secondary antibodies for 1 h at room temperature. Immunoreactivities were revealed with the ECL Western Blotting Reagent (Amersham). Quantifications were performed with the operating system ChemiDoc (Biorad) and analyzed with the software ImageLab (Biorad). Densitometric values from murine caspases 1, 8 and 11 antibodies on kidney extracts were normalized by the anti-β actin (Monoclonal Anti-β-Actin antibody produced in mouse, A5316, Sigma) signal intensity, and then normalized to sham.

#### Histologic analysis

Kidneys were stored in Carnoy solution (ethanol 60%, chloroform 30%, acetic acid 10%) for 48 h and then in 70% alcohol. They were dehydrated and embedded in paraffin and 4 µm paraffin-embedded tissue sections were cut and used for immunohistochemistry and histological staining.

#### TUNEL

Cell death was studied through the TUNEL (Terminal Deixynucleotidyl Transferase dUTP Nick End Labelling) method using Apoptotag Peroxidase Det Kit (S7100, Merkmillipore). Sections were scanned using a Nanozoomer 2.0 RS (Hamamatsu Photonics SARL, Massy, France) and quantification was performed manually by counting nuclei in the cortex and cortico-medullary junction. Then the account was reported to the area of the studied field.

#### Immunohistochemistry for collagen III

As previously described^42^, after endogenous peroxidase blockade (S2001, DakoCytomation) tissue sections were incubated for 1 h with primary antibody anti-collagen type III (Rabbit anti Mouse/Rat Collagen type III alpha 1 chain, Acris Antibodies GmbH) and then for 1 h with the secondary anti-rabbit IgG (Dako Envision HRP system). Immunological complexes were visualized by the addition of the DAB substrate during 10 min (TA-125-HDX, Thermo Fisher Scientific). Sections were counterstained with hematoxylin. Sections were scanned using a Nanozoomer 2.0 RS (Hamamatsu Photonics SARL, Massy, France) and treated with ImageJ analysis software for morphometric analyses.

#### Statistical analyses

Statistical comparisons used a Kruskall-Wallis with a Dunn post-hoc test, performed using GraphPad Prism Version 6. Difference between groups were considered significant when *P* < 0.05. Data were expressed as mean ± standard error of the mean (m ± s.e.m.).

## Supplementary information

Supplementary Information
