# Supplementary material for: A single dose of estrogen during hemorrhagic shock protects against Kidney Injury whereas estrogen restoration in ovariectomized mice is ineffective
Source: Sci Rep. 2020 Oct 14;10:17240. doi: 10.1038/s41598-020-73974-5 (PMC7560623; doi:10.1038/s41598-020-73974-5)
Supplement: Supplementary file 1 — Supplementary Information [file 41598_2020_73974_MOESM1_ESM.pdf]

A single dose of estrogen during hemorrhagic shock protects against Kidney Injury whereas estrogen restoration in ovariectomized mice is ineffective.

Marie Buléon [1,2*], Mélodie Cuny [1,2*], Jimmy Grellier [1,2], Pierre-Yves Charles [1,2], Julie Belliere [1,2], Audrey Casemayou [1,2], Jean-François Arnal [1,2], Joost-Peter Schanstra [1,2], Ivan Tack [1,2]

1 Institut National de la Santé et de la Recherche Médicale (INSERM), U1048, Institute of Cardiovascular and Metabolic Disease, Toulouse, France

2 Université Toulouse III Paul-Sabatier, Toulouse, France

* These authors contributed equally to this work

Corresponding author: Ivan TACK
Address: Service des Explorations Fonctionnelles Physiologiques, CHU de Toulouse, 1 avenue Jean Poulhès – 31 059 Toulouse Cedex 9 - France
Tél: +33-562-889-097
Fax: +33-561-322-801
Email: ivan.tack@univ-tlse3.fr

Supplementary informations

<u>Supplementary figure 1</u>: Full-lenght blot of caspase 8 presented in Figure 4c

Caspase 8 (p18)

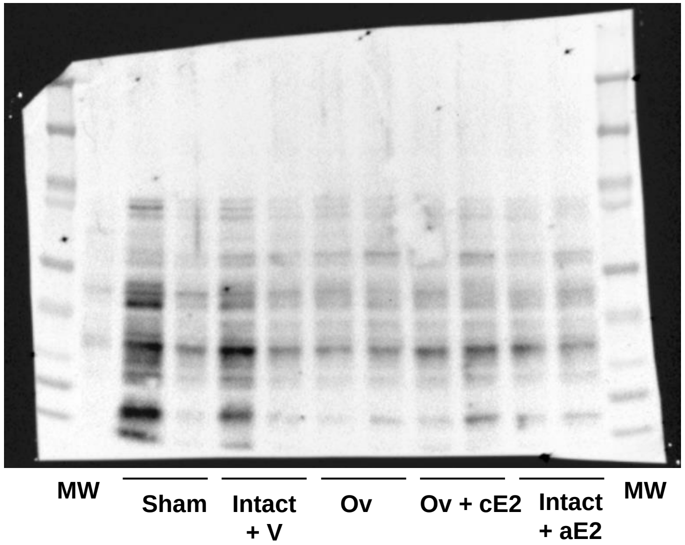

β Actin

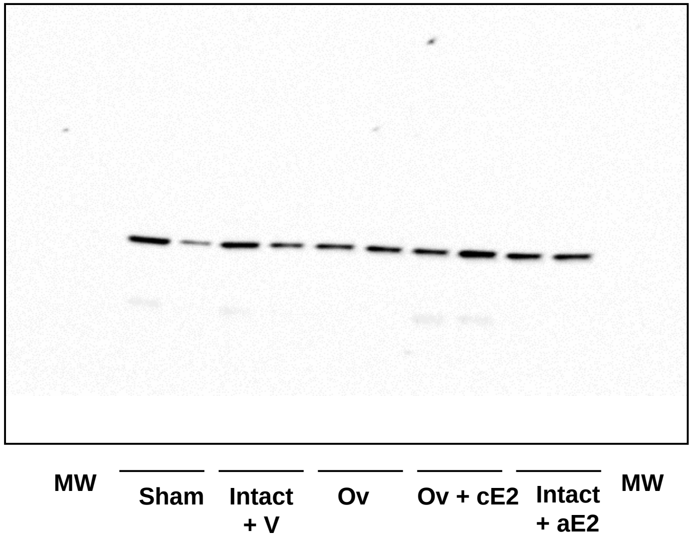

Presented in the manuscript (figure 4c) as :

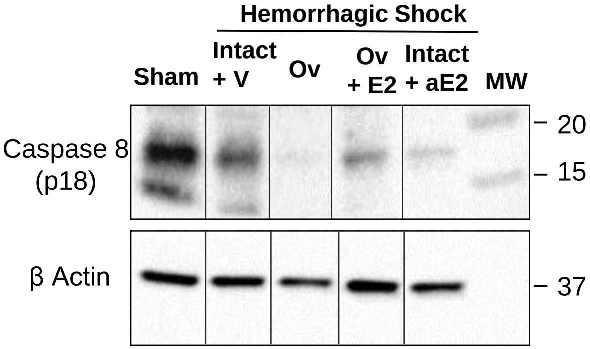

<u>Supplementary figure 2</u>: Full-lenght blot of caspase 1 presented in Figure 5d

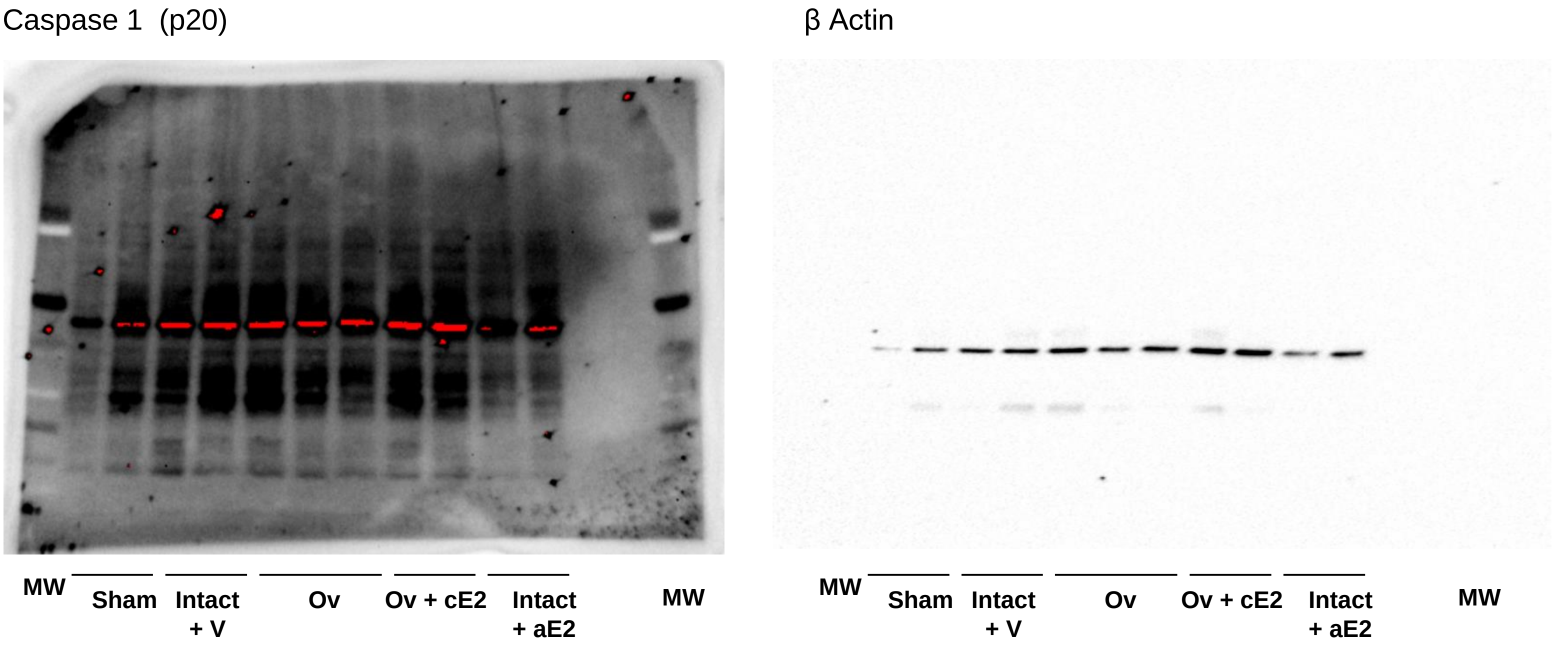

Presented in the manuscript (figure 5d) as :

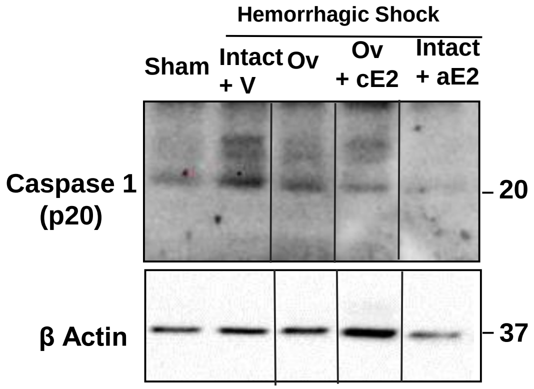

Supplementary figure 3: Full-lenght blot of caspase 11 presented in Figure 6d

Caspase 11 (p20)

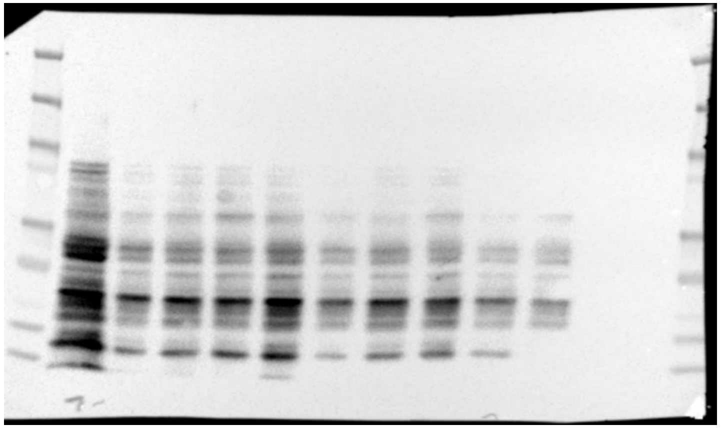

MW Sham Intact + V Ov Ov + cE2 Intact + aE2 MW

β Actin

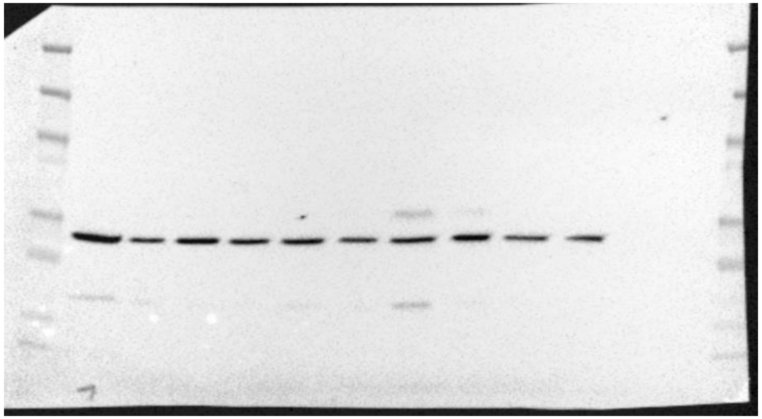

MW Sham Intact + V Ov Ov + cE2 Intact + aE2 MW

Presented in the manuscript (figure 6d) as :

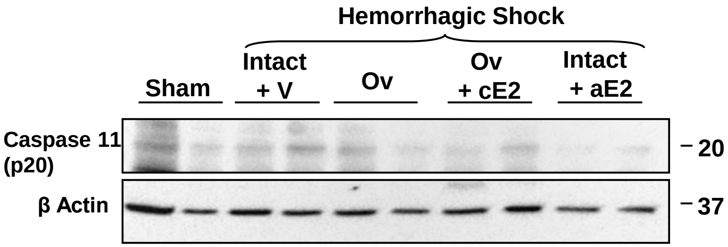

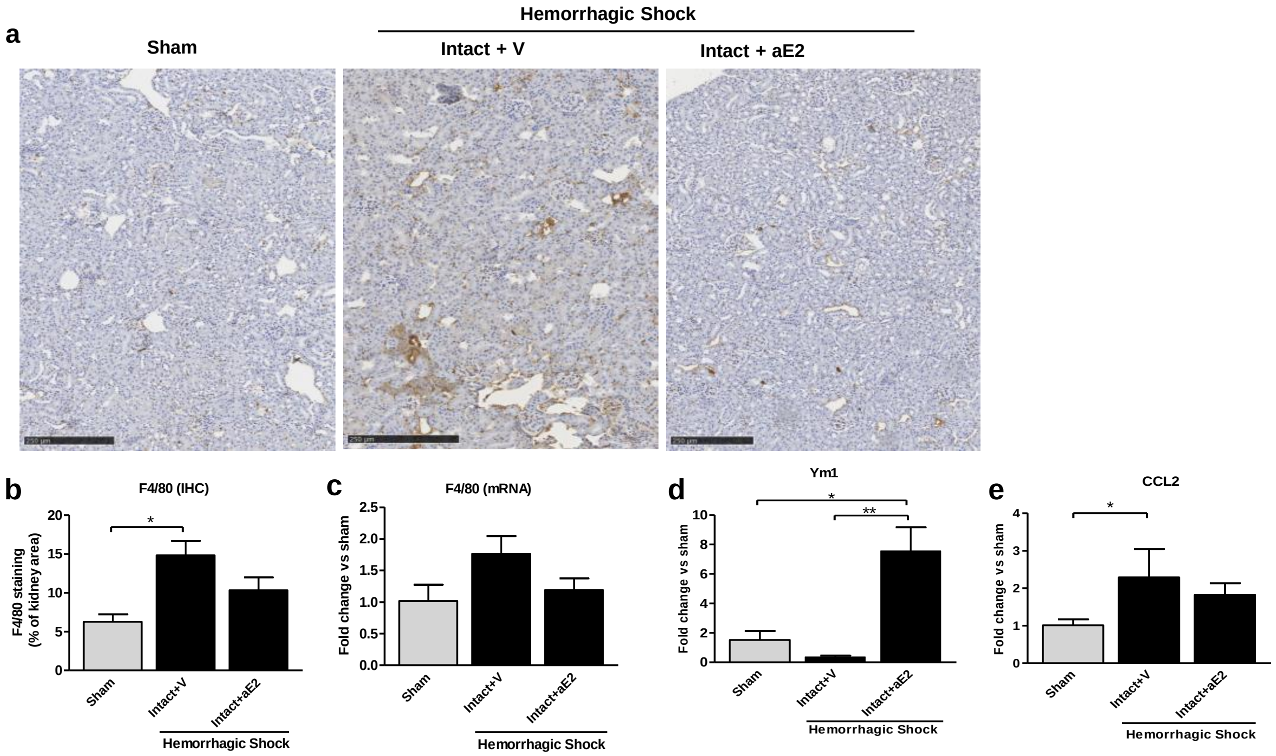

Supplementary Figure 4. Macrophage recruitment one day after hemorrhagic shock: **a) Representative Pictures of F4/80 immunostaining on kidney sections** (magnification x400, counterstaining with HE); **b) F4/80 expression** (immunostaining analysis), *n=5 in all groups*; **c) EmR1** (=F4/80) mRNA expression; **d) Ym1** (M2 macrophage marker) mRNA expression; **e) CCL2** mRNA expression. *For EmR1, Ym1 and CCL2 mRNA expression: n=6 in all groups. Sham: non-shocked mice; Intact + V: intact mice treated by vehicle (NaCl); Intact + aE2: Intact mice receiving a unique administration of 25 µg estradiol at the end of resuscitation; Intact + V and Intact + aE2 groups are submitted to hemorrhagic shock*. Data are presented as mean ± s.e.m.* P<0.05 ; ** P<0.01 for indicated comparison.

Comment : Since acute renal ischemia also involves macrophages at the time of damages and repair, their markers of recruitment were studied one day after shock. In control group, shock induced a mild change in these markers that was partly prevented by the bolus of estradiol as shown by F4/80 staining (Supplementary Figure 4a, b and c). Shock was also associated with an increase in the chemoattractant CCL2 (Supplementary Figure 4e). In the group that has received the bolus of estrogen at the end of shock, a recruitment of YM1 mRNA expression, a marker associated with the M2 phenotype, was observed (Supplementary Figure 4d).

These preliminary results indicate that the bolus of estradiol was associated with a differential renal macrophage expression one day following shock. A differentiation of renal macrophages toward a M2 profile would potentially be associated with a repairing activity. This preliminary observation requires further work to establish the direct effect of estradiol on both tubular cells and macrophages infiltration in this situation.
